# Catheter-related bloodstream infection rates: Comparing cuffed vs. uncuffed catheters in a nationwide series of small children on chronic hemodialysis 

**DOI:** 10.5414/CNP104S07

**Published:** 2025-11-28

**Authors:** Gregor Novljan, Rina R. Rus, Nina Battelino

**Affiliations:** 1 Pediatric Nephrology Department, Children’s Hospital, University Medical Center Ljubljana, and; 2 Faculty of Medicine, University of Ljubljana, Ljubljana, Slovenia

**Keywords:** hemodialysis, catheter, infection, children

## Abstract

Introduction: Catheter-related bloodstream infections (CBSI) are serious complications in pediatric hemodialysis (HD) patients. We aimed to compare the CBSI rates associated with cuffed and uncuffed central venous catheters (CVC) in small children. Materials and methods: All HD patients weighing < 15 kg and dialyzed via cuffed CVCs for at least 3 months between March 2016 and March 2022 were included. The CBSI rate was compared to that of a well-matched historical series of our patients before implementing cuffed CVCs. Results: Three boys and 1 girl (median weight: 14.0 kg) matched the inclusion criteria and received HD using the same type of cuffed CVC. Eleven CBSIs occurred during 4,870 days with cuffed CVCs, yielding a CBSI rate of 2.3/1,000 catheter days, compared to 7.7/1,000 catheter days in our historical series with uncuffed CVCs (p = 0.002). A 70% reduction in the CBSI rate was achieved with cuffed CVCs (p = 0.002). The median catheter survival times for cuffed and uncuffed CVCs were 189 and 53 days, respectively (p = 0.002). Conclusion: Our results show that cuffed CVCs are associated with reduced CBSI rates and improved catheter longevity compared to uncuffed ones in small children.

## Introduction 

Kidney transplantation is the optimal treatment for children with end-stage kidney disease (ESKD). However, due to the patient’s size or complicated conditions, a period of bridging dialysis is often needed before transplantation. Peritoneal dialysis (PD) is preferred in infants and young children but may be limited or even contraindicated, and chronic hemodialysis (HD) becomes the only treatment alternative [1, 2]. 

Proper vascular access is critical for successful HD. Despite the “Fistula First” initiative [3], and according to available data, the majority of pediatric patients (55 – 84%) still initiate HD with a central venous catheter (CVC) [4, 5, 6, 7]. Creating an arterio-venous fistula (AVF) in pediatric patients is challenging due to age-related anatomical limitations, particularly in children weighing < 20 kg [8]. CVCs, in turn, are associated with much higher complication and access failure rates than AVFs [5, 9]. 

In a previously published study [10], we analyzed our experiences with chronic HD in a series of small children using exclusively uncuffed CVCs. Catheter-related bloodstream infections (CBSI) were the most frequent complication, and the CBSI rate of 7.7 episodes per 1,000 catheter days was higher than that in the existing literature [10, 11, 12, 13, 14, 15]. An initiative was undertaken to change concepts. Consequently, tunneled cuffed CVCs became the standard of care for children unfit for AVF formation in our unit, as suggested by the European Society for Pediatric Nephrology Dialysis Working Group [16]. 

In the present study, we aimed to evaluate the impact of implementing new catheter selection practices and compare the outcomes, particularly the CBSI rates associated with cuffed and uncuffed CVCs in children weighing < 15 kg on maintenance HD in our country. 

## Materials and methods 

All Slovenian small children unsuitable for PD or AVF formation and receiving maintenance HD for more than 3 months from March 2016 to March 2022 were included in the study. Anatomical limitations for HD related primarily to the patient’s size rather than chronological age, and a significant proportion of children with ESKD present with growth issues. We used weight as an inclusion criterion, with “small children” referring to patients weighing < 15 kg. This approach was consistent with our previous study [10] and has also been utilized by other authors [12, 13, 14]. 

**Figure 1:**
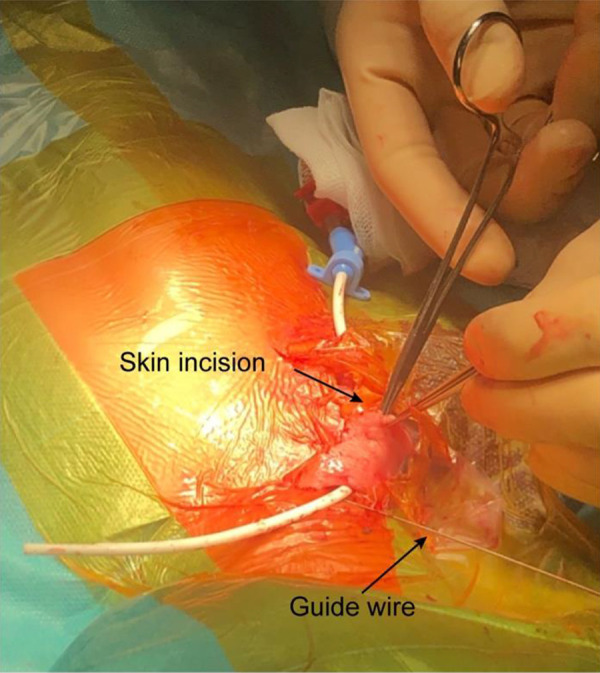
Catheter insertion. Insertion of a cuffed dialysis catheter with minimally invasive surgery. One or two small incisions in the skin are made for optimal subcutaneous catheter placement. The catheter tip is railroaded over the guide wire, previously inserted percutaneously into the right atrium using the Seldinger technique and ultrasound guidance.

Double-lumen cuffed silicone 8F or 10F 18-cm-long catheters (Hemo-Cath LT, Medcomp Inc., Harleysville, PA, USA) were used in all cases. A dedicated cardiovascular surgeon placed all catheters using minimally invasive techniques under general anesthesia (Figure 1). Ultrasound guidance and intraoperative fluoroscopy were used for optimal catheter tip positioning. The right jugular vein was preferred, but the left one was used if a previous catheter had to be removed and guide-wire exchange was contraindicated. Subclavian veins were not used for catheter insertion. Meticulous care was given to avoid kinking of the subcutaneous part of the catheter. 

Catheter handling was performed by trained, specialized dialysis nurses, strictly adhering to aseptic precautions according to recommended care practices [17]. Catheter use was restricted to HD with clearly defined exceptions, including antibiotic administration on dialysis days and intradialytic blood transfusions for children with volume issues. Topical antibiotic prophylaxis with mupirocin was used, but was temporarily changed to comply with swab results. Catheter locking was performed exclusively with tissue plasminogen activator (t-PA) (i.e., alteplase). 

Intermittent HD was performed using the Prismaflex system (Baxter, Deerfield, IL, USA) or the 5008 CorDiax dialysis machine (Fresenius, Bad Homburg, Germany), with 3 – 4 weekly HD sessions. Citrate anticoagulation was initially used in all patients. Heparin was eventually introduced in 1 patient. The blood flow rates ranged from 60 to 110 mL/min (5 – 8 mL/kg/min) and were often restricted due to catheter issues. Initially, the same dialyzer was used in all patients: the Prismaflex ST-60 (AN69 membrane) marketed by Baxter (filter surface area: 0.6 m^2^; priming volume: 93 mL). Two patients were subsequently switched to the 5008 CorDiax machine using an FX 40 or FX 50 dialyzer (Helixone membrane; Fresenius) with a filter surface area of 0.6 m^2^ and 1.0 m^2^, respectively. 

CBSI was defined as fever, with other possible causes excluded, elevated inflammatory markers, and a positive blood culture. Blood cultures were predominantly taken from both catheter hubs. In some cases, additional blood cultures were taken from the peripheral vein. One patient was treated for suspected CBSI following our unit’s protocol and was included in the statistical analysis despite a negative blood culture, as the clinical manifestations were highly indicative of CBSI. 

Statistical analysis was performed using IBM SPSS for Macintosh (version 24.0, IBM Corp, Armonk, NY, USA). Patient demographics, including age and body weight at cuffed catheter insertion, catheter type, insertion site, catheter longevity, and catheter-related complications, were collected retrospectively by reviewing the patients’ medical records and the microbiology database. The CBSI rate, calculated as incidence per 1,000 catheter days, represented the primary outcome. Microbiology results and treatment outcomes were also registered. Data were analyzed using descriptive statistics, including frequencies, percentages, and median values with range for categorical variables. 

The results of the current patient series were then compared with a well-matched historical control series of patients who had been dialyzed in our unit before cuffed catheters were introduced [10]. No differences regarding dialysis equipment and catheter handling existed between the groups. The Shapiro-Wilk’s test, visual inspection of histograms, and box plots were used to test for normal distribution of variables. The Mann-Whitney test for independent nonparametric variables and Pearson’s χ^2^-test for independent frequency variables were used to check for significant differences between groups, as appropriate. A p-value < 0.05 was considered statistically significant. 

The Institutional Review Board and the National Medical Ethics Committee (No. 0120-176/2025-2711-4) approved all procedures in this study. Informed consent was received from all participants. 

## Results 

All 4 eligible Slovenian patients (1 girl and 3 boys) were analyzed. The median age and median weight at initial cuffed CVC placement were 4.1 years (2.9 – 10.2 years) and 14.0 kg (12.4 – 17.1 kg). In all patients, HD was the initial dialysis modality. The reasons for choosing HD over PD were peritoneal membrane failure, peritoneal adhesions due to previous abdominal surgery, socio-economic background with language issues, and parents’ choice. A comparison of current demographic data with our historical series is shown in Table 1. No statistically significant differences were found between the groups regarding patient number, gender distribution, median age and median body weight at initial catheter insertion (U = 4.00, z = –1.16, p = 0.343 and U = 3.00, z = –1.44, p = 0.200, respectively). The median patient weight was at the 8^th^ percentile for age (Z-score: –1.42). 

The reasons for kidney failure were cystic dysplastic kidneys associated with Hajdu-Cheney syndrome, infantile nephrotic syndrome (*WT-1* mutation), lupus nephritis with hypocomplementemic urticarial vasculitis and positive ANCA (*DNASE1L3* mutation), and chronic glomerulopathy after thymic transplantation due to CHARGE syndrome. 

During the study, 16 long-term cuffed CVCs were used, with 4 initial insertions and 12 revisions. The cumulative catheter usage time was 4,870 days (i.e., 13.3 patient-years). Catheter survival was defined as the number of catheter days from insertion until removal at the completion of HD therapy, catheter revision for malfunction or infection, or conclusion of the study with a still functioning catheter. The median overall catheter survival, combining mechanical and infectious reasons for catheter removal, was 189 days (13 – 1,120 days), as compared to 53 days (6 – 373) in our historical series (*U* = 71.00, z = –2.97, p = 0.002). Catheter usage and replacement rates are outlined in Table 2. 

**Figure 2:**
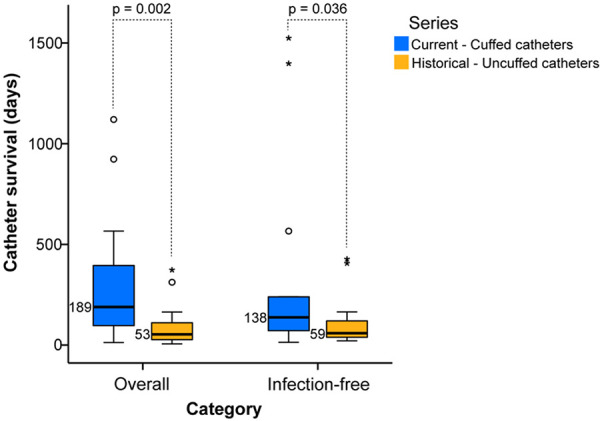
Comparison of catheter survival. Box and whiskers plot showing the median, quartile, and extreme values of overall and infection-free catheter survival times, stratified by series-specific catheter type. The boxes represent the interquartile range (IQR). Some outliers can be seen and are related to the non-normal data distribution. The horizontal lines in the boxes indicate median values. The difference between median cuffed (blue boxes) and uncuffed (orange boxes) catheter survival was significant in both overall (p = 0.002) and infection-free survival (p = 0.036).

Eleven CBSIs were recorded during the observation period. The median CBSI rate was 2.3/1,000 catheter days, representing a 70% reduction from the 7.7 rate with uncuffed catheters in our historical series, achieving statistical significance (χ^2^ = (1, N = 8) = 9.20, p = 0.002). Infection-free survival (i.e., time-span without the occurrence of CBSI) increased to a median of 138 days (14 – 1,524 days) compared to 59 days (21 – 427 days) in the historical series, which is also significantly longer (U = 77.00, z = –2.10, p = 0.036). Only 5/11 (46%) cuffed CVCs were replaced due to CBSIs, compared to 11/14 (78%) uncuffed catheters in the historical series (Figure 2). 

Gram-positive organisms were isolated in 9/16 (56%) blood cultures. The *Streptococcus* and *Staphylococcus* species were the most frequently found Gram-positive organisms (7/9). Gram-negative bacteria were present in 6/16 (38%). The *Pseudomonas* species and the closely related *Stenotrophomonas maltophilia* and *Serratia marcensens* were the most often isolated Gram-negative species (4/6). More than one organism was isolated in 4/11 (36%) CBSI episodes. CBSIs caused by fungal agents were not registered. On 1/11 (9%) occasions, the blood cultures remained sterile. A detailed distribution of microbiology results is presented in Table 3. 

A total of 12 catheter revisions occurred, with 42% (5/12) due to infections and 58% (7/12) related to catheter malfunctions such as luminal blockage, catheter tip malposition, or kinking of the subcutaneous catheter portion. One catheter required urgent replacement due to mechanical damage during dressing removal. The overall revision rate was 0.9/patient-year or 2.5/1,000 catheter days. Specifically, in 46% (5/11) of cases with CBSI the catheters were replaced compared to 79% (11/14) in the historical series. The difference was not statistically significant (χ^2^ = (1, N = 25) = 1.67, p = 0.196). Detailed comparisons can be found in Table 2. 

Two patients received their first cuffed catheter in the right jugular vein, which is the preferred site. For the other 2 patients, left jugular vein insertions were necessary due to a previous infected CVC in the right jugular vein requiring catheter exchange in 1 case and, in the other case, due to a pre-existing port implanted in the right jugular vein combined with challenging anatomy on that side, according to ultrasound vein mapping. Details are in Table 1. 

## Discussion 

Our study contributes valuable data to the limited evidence comparing CBSI rates between cuffed and uncuffed catheters in children. We present the results of implementing strictly cuffed instead of uncuffed catheter use in a nationwide series of small children on HD. This conceptual change was triggered by our previously published outcome analysis, stressing the urgent need for improvements to minimize CBSI risk, as our historical CBSI rate in children weighing < 15 kg compared unfavorably to existing literature [10]. 

Historically, a CBSI rate of 1 – 2/1,000 catheter days has been regarded as good, while CBSI rates of 1/1,000 catheter days were considered excellent in adult patients using cuffed dialysis catheters, with a steady trend of improvement in recent years [18, 19]. Findings from the International Pediatric Hemodialysis Network (IPHN) Registry, which involved the prospective follow-up of 552 children with cuffed CVCs, revealed an overall infection rate of 1.3/1,000 catheter days. CBSIs accounted for 73% and exit site or tunnel infections for 27% [5]. 

As observed in a previous analysis, the CBSI rate in our unit’s overall pediatric HD population, with a median age of 13.3 years (range: 2.5 – 17.7) at HD initiation, was 0.9/1,000 catheter days, despite the use of uncuffed catheters with citrate lock [20]. This prompted us to conclude that uncuffed short-term CVCs may be an acceptable alternative for long-term HD vascular access in children unfit for AVF formation. Additionally, such catheters are easily inserted and replaced using a guide wire. However, the findings of our current study indicate that this does not apply to infants and small children. 

Pediatric studies comparing CBSI rates between cuffed and uncuffed CVCs are limited and characterized by small sample sizes and short-term outcomes. However, a recent study analyzed 19 children starting HD in their first year of life, finding that cuffed catheters significantly reduced CBSI rates compared to uncuffed ones (0.9 vs. 9.3/1,000 catheter days) [21]. Another retrospective study of 187 children aged 4.5 – 12 years in a middle-income country showed CBSI rates of 25.8 vs. 10.1/1,000 catheter days for uncuffed and cuffed CVCs, respectively [22]. Cuffed CVCs are typically recommended in specific circumstances, if HD treatment is anticipated to last longer than 14 days [16, 23]. 

In our current study, implementing cuffed instead of uncuffed CVCs for chronic HD in small children caused an impressive 70% reduction in CBSI rates (2.3 vs. 7.7/1,000 catheter days) (p = 0.002). The increased risk of infection at a young age is multifactorial. Strict adherence to HD catheter care standards is crucial for preventing catheter-related infections [17]. Since patient characteristics, dialysis procedures, catheter care, and handling did not differ significantly between our current and historical patient series, it can be reasonably assumed that the improved CBSI rate may be attributed to cuffed catheter usage. 

The median overall catheter survival time, including mechanical and infectious causes for catheter revision, was significantly longer for cuffed than uncuffed CVCs in our study (189 vs. 53 days). This was also true for the median infection-free catheter survival, with catheters not being replaced at each occurrence of CBSI (138 vs. 59 days). Feinstein et al. [24] reported a mean overall catheter survival time of 5.7 months (171 days) for cuffed and 1.3 months (39 days) for uncuffed catheters in 20 dialyzed infants. Another similar study examining the outcomes in 29 infants demonstrated a mean overall usage time of 139 vs. 7.2 days for cuffed and uncuffed catheters [21]. Given the small sample size and non-normal distribution, we opted to present our results using the median value. If we had used the mean value, as reported by Feinstein et al., the overall survival time would have been 304 days. Our findings are consistent with existing literature on using cuffed HD catheters in small children (i.e., 21 – 390 days) [11, 12, 13, 14, 15, 21, 25, 26]. 

Compared to our historical series, the CBSI rates decreased, while mechanical complications increased with cuffed CVCs. Catheter malfunction necessitated replacements in 58% compared to 27% in our historical series. The prevalence of mechanical issues with cuffed CVCs in small children is well documented [11, 12, 13]. Pollack et al. [26] reported notably low infection and malfunction rates in 18 infants using cuffed catheters for HD, with 50% removed due to malfunctions and none for infections. Lopez et al. [14] found that mechanical issues were the main reason for removing cuffed catheters in 11 children weighing < 15 kg, accounting for 39%, while infections comprised 13%. 

Limitations of our study include a retrospective design and small sample size, limiting the scope and strength of the statistical analysis. It is common practice to prefer PD for chronic dialysis treatment of pediatric patients younger than 5 years [1]. HD is used when PD fails or is inapplicable and was reported as the initial dialysis modality in only 6% of cases in a cohort of children under 2 years of age [12, 15]. It is, therefore, not surprising that only a limited number of predominantly single-center reports on HD in these patients exist. Thus, the small number of patients in our current study is expected to reflect the low prevalence and incidence rate of such patients in our country. Notably, all Slovenian children are dialyzed in our unit. Furthermore, a historical control group was used in the study to replace a concurrent control. This approach was taken because it seemed unwarranted to recruit patients for a control arm, as the results of our previous work undoubtedly pointed to the urgent need to change settled practices. Also, the already small patient number would have been additionally diminished. Selection bias was omitted, as all candidate patients were enrolled in the study in both series. Systematic differences among the current and historical groups could be excluded, as there were no significant disparities in patient demographics, and both groups were treated in the same healthcare environment, applying the same standards of care. The study designs were equal, and endpoints were clearly defined, as were definitions of diagnosis and measurement. All these considerations have been shown to minimize the disadvantages of historical controls [27]. 

In conclusion, despite using preventive measures and cuffed HD catheters, infections remain common in small children. Although the results of our study are significant, showing a clear statistical advantage in using cuffed over uncuffed catheters in reducing CBSI rates and improving catheter longevity, our findings should be interpreted with caution due to the small sample size. Further efforts are needed to improve patient outcomes. 

## Compliance with ethical standards 

The institutional review board and the National Medical Ethics Committee approved all procedures in this study (No. 0120-176/2025-2711-4). All patients gave written informed consent before their inclusion in the study. 

## Data availability statement 

The supporting data and findings of this study are available from the corresponding author upon reasonable request. 

## Authors’ contributions 

Conceptualization, G.N. and N.B.; methodology, G.N. and N.B.; acquisition of patient data, N.B. and R.R.R.; validation, G.N.; formal analysis, G.N.; writing – original draft preparation, G.N.; writing – review and editing, all authors; visualization, G.N. All authors read and approved the final version of the manuscript. 

## Funding 

This work received no grant from public, commercial, or not-for-profit funding agencies. 

## Conflict of interest 

All authors declare that they have no competing interests. 

**Table 1.** Comparison of patient and catheter characteristics.

		Series		p-value*
		Current (N = 4)	Historical (N = 4)	
Gender (N (%))	Girls	1 (25%)	1 (25%)	–
	Boys	3 (75%)	3 (75%)	–
Age at catheter insertion	Median (years)	4.1	2.8	0.343
	Range (years)	(2.9 – 10.2)	(2.3 – 7.7)	–
Weight at catheter insertion	Median (kg)	14.0	11.8	0.200
	Range (kg)	(12.4 – 17.1)	(11.4 – 15.1)	–
Initial catheter type	Cuffed long-term double-lumen 8F × 18 cm	4	0	–
	Uncuffed pre-curved double-lumen 8F × 12 cm	0	2	–
	Uncuffed straight double-lumen 8F × 12.5 cm	0	2	–
Initial insertion site	Right jugular vein	2	2	–
	Left jugular vein	2	0	–
	Right subclavian vein	0	2	–
	Left subclavian vein	0	0	–

*Mann-Whitney test exact significance [2*(1-tailed Sig.)].

**Table 2.** Comparison of catheter-related infections, catheter survival, and revision rates.

		Series		p-value
		Current	Historical	
Total number of catheters used		16	21	–
Cumulative catheter days		4870	1810	–
Number of CBSI		11	14	–
CBSI rate (N/1,000 catheter days)		2.3	7.7	0.002^a^
Catheter revisions due to CBSI (N (%))		5 (46%)	11 (79%)	–
Overall catheter survival	Median (days)	189	53	0.002^b^
	Range (days)	(13 – 1120)	(6 – 373)	–
Infection-free catheter survival	Median (days)	138	59	0.036^b^
	Range (days)	(14 – 1524)	(21 – 427)	–
Overall catheter revisions	Infection (N (%))	5 (42%)	11 (73%)	–
	Malfunction (N (%))	7 (58%)	4 (27%)	–
	Total (N (%))	12 (100%)	15 (100%)	–
Overall catheter revision rate (N/patient-year)		0.9	3.0	–

CBSI = catheter-related bloodstream infection. ^a^Pearson’s χ^2^-test with Yates’ continuity correction. ^b^Mann-Whitney test Exact Significance [2*(1-tailed Sig.)].

**Table 3.** Microbiology results.

		Case count (N = 16)	Subtable (%)	Table (%)
Gram-positive bacteria	Streptococcus pneumoniae	2	22%	13%
	Staphylococcus pseudointermedius	2	22%	13%
	Streptococcus pyogenes	1	11%	6%
	Staphylococcus epidermidis	1	11%	6%
	Staphylococcus aureus	1	11%	6%
	Micobacterium arborescens	1	11%	6%
	Enterococcus faecalis	1	11%	6%
	**Subtable total**	**9**	**100%**	**56%**
Gram-negative bacteria	Serratia marcensens	2	33%	13%
	Stenotrophomonas maltophilia	1	17%	6%
	Pseudomonas aeruginosa	1	17%	6%
	Ochrobactrum anthropi	1	17%	6%
	Delfia acidovorans	1	17%	6%
	**Subtable total**	**6**	**100%**	**38%**
Negative culture	No causative organism identified	1	100%	6%*
	**Table total**	**16**	**100%**	**100%**

*Of note: 6% (1/16) relates to all isolates. Based on the number of catheter-related bloodstream infections (including polymicrobial cases), the negative culture rate was 9% (1/11).
